# Myokines and Their Potential Protective Role Against Oxidative Stress in Metabolic Dysfunction-Associated Steatotic Liver Disease (MASLD)

**DOI:** 10.3390/antiox13111363

**Published:** 2024-11-07

**Authors:** José Luis Bucarey, Isis Trujillo-González, Evan M. Paules, Alejandra Espinosa

**Affiliations:** 1 School of Medicine, Faculty of Medicine, Universidad de Valparaíso, San Felipe 2172972, Chile; jose.bucarey@uv.cl; 2 Nutrition Research Institute, Gillings School of Public Health, University of North Carolina at Chapel Hill, Chapel Hill, NC 27599, USA; itruji@email.unc.edu (I.T.-G.); paulese@unc.edu (E.M.P.); 3 Center of Interdisciplinary Biomedical and Engineering Research for Health, Universidad de Valparaíso, San Felipe 2172972, Chile

**Keywords:** MASLD, GPX4, lipid peroxidation, ferroptosis, myokines, oxidative stress

## Abstract

Myokines, bioactive peptides released by skeletal muscle, have emerged as crucial regulators of metabolic and protective pathways in peripheral tissues, particularly in combating oxidative stress and inflammation. Their plasma concentration significantly increases following exercise, offering valuable insights into the role of physical activity in preventing sarcopenia and mitigating metabolic diseases, including obesity, diabetes, and metabolic dysfunction-associated steatotic liver disease (MASLD). This review focuses on discussing the roles of specific myokines in activating intracellular signaling pathways within the liver, which confer protection against steatosis and lipid peroxidation. We detail the mechanism underlying lipid peroxidation and highlight the liver’s antioxidant defenses, such as glutathione (GSH) and glutathione peroxidase 4 (GPX4), which are pivotal in reducing ferroptosis. Furthermore, we provide an in-depth analysis of key myokines, including myostatin, brain-derived neurotrophic factor (BDNF), and irisin, among others, and their potential impact on liver function. Finally, we discuss the molecular mechanisms through which these myokines influence oxidate stress and lipid metabolism, emphasizing their capacity to modulate antioxidant responses in the liver. Finally, we underscore the therapeutic potential of exercise as a non-pharmacological intervention to enhance myokine release, thereby preventing the progression of MASD through improved hepatic antioxidant defenses. This review represents a comprehensive perspective on the intersection of exercise, myokine biology, and liver health.

## 1. Introduction

Obesity is a major driver of metabolic dysfunctions, including metabolic dysfunction-associated steatotic liver disease (MASLD). The global prevalence of MASLD is around 30% of the global population [1]. MASLD encompasses a range of liver diseases, such as non-alcoholic steatohepatitis (NASH), characterized by inflammation and liver damage that can lead to scarring [2]. A key factor for the development of these conditions is oxidative stress, which propels the accumulation of membrane lipid peroxides in cellular membranes [3]. Lipid droplets (LDs) within the hepatocyte play a protective role in preventing lipoperoxidation and protecting the plasma membrane from the harmful effects of oxidation of polyunsaturated fatty acids (PUFAs). When there is a buildup of lipid peroxides, the cell initiates ferroptosis, an iron-dependent programmed cell death that is mostly caused by the process of membrane lipoperoxidation [4]. In ferroptosis, iron serves as a catalyst, and mitochondria experience a reduction in volume while the cell membranes remain intact (details on the mechanisms of ferroptosis will be further discussed below). Importantly, in liver injury and MASLD, liver cells are more susceptible to ferroptosis [5].

The renaming of NAFLD to MASLD reflects an evolving understanding of the disease etiology, emphasizing the centrality of metabolic dysfunction, particularly insulin resistance (IR). Exercise, recognized as one of the most effective treatments for MASLD, reduces fatty tissue and liver fat infiltration, helping to mitigate oxidative damage and inflammation and even improve fibrosis scores associated with disease progression [6]. During exercise, myokines are produced by skeletal muscle cells and play a key role in metabolic health and inter-organ crosstalk. They influence physiological processes such as insulin sensitivity, aging, glucose metabolism, and pancreatic beta-cell function, impacting various organs [7,8]. This review provides a complementary perspective on the liver-protective effects of muscle-derived myokines, emphasizing their role in preventing lipoperoxidation and ferroptosis in MASLD.

## 2. Insulin Resistance as a Central Driver in MASLD

A hallmark of metabolic syndrome is IR, which also plays a significant role in the development of MASLD. IR occurs when cells in muscle and adipose tissue become less responsive to insulin, a hormone essential for maintaining glucose homeostasis [9,10]. In response, the pancreas produces more insulin, as higher levels are needed to regulate blood glucose effectively. In the liver, IR leads to increased de novo lipogenesis and reduces fatty acid oxidation, causing an accumulation of triglycerides inside the hepatocytes [11]. Lipid overload is one of the major factors of hepatic steatosis, a hallmark of MASLD. The progression from hepatic steatosis to more severe forms of liver diseases, such as non-alcoholic steatohepatitis NASH, involves several factors, with IR playing a central role [12]. IR leads to hyperglycemia and hyperinsulinemia, both of which contribute to increased oxidative stress and inflammation [13,14]. This harmful environment triggers the peroxidation of LDs, resulting in the formation of reactive aldehydes like malondialdehyde (MDA). These toxic byproducts cause damage to cellular components, leading to inflammatory responses and fibrosis.

The role of ferroptosis adds further complexity to this scenario. IR is often accompanied by low-grade inflammation, which raises levels of inflammatory cytokines. These cytokines may trap iron within hepatic stellate cells (HSCs), leading to an imbalance where iron overload in HSCs and iron deficiency in hepatocytes are key features of both steatohepatitis and NAFLD [15,16]. This imbalance triggers ROS-dependent activation of the iron-induced fibrogenic processes in HSCs [17]. Additionally, research suggests that chronic iron overload associated with IR may worsen it by promoting hepatic ferroptosis via inhibition of the JAK2/STAT3/SLC7A11 pathway [8]. Treatment with the iron chelator deferasirox has been shown to improve hepatic IR caused by iron overload [8].

In the context of IR, the liver’s antioxidant defenses, including GSH and GPX4, are insufficient to counteract redox imbalance [18,19]. This imbalance leads to the accumulation of lipid peroxides, which triggers necroptotic cell death and exacerbates liver inflammation and damage [20]. Aerobic exercise primarily relies on oxidative metabolism and stimulates type I (slow twitch) muscle fibers, which are rich in mitochondria and well-suited for endurance activities. In contrast, resistance exercise activates glycolytic metabolism and engages type II (fast-twitch) muscle fibers, specialized for short bursts of power and strength [21,22]. Notably, resistance exercise has been found to improve NAFLD more effectively than aerobic exercise, likely due to the release of myokines that may positively influence lipid metabolism in the liver [23].

## 3. Mechanisms of Lipid Peroxidation

Lipoperoxidation is a process by which oxidants attack lipids, mostly PUFAs, in a cell membrane. This reaction can propagate through additional membranes in a chain reaction, which can only be halted by an antioxidant.

### Lipoperoxidation Process

**Figure 1:**
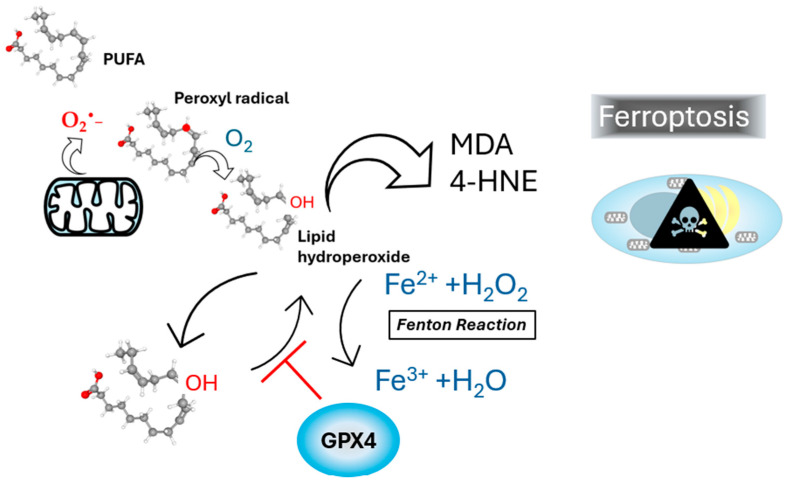
Lipoperoxidation and ferroptosis. Polyunsaturated fatty acids (PUFAs), such as linolenic acid, are prone to oxidation when interacting with molecular oxygen (O_2_), leading to the formation of lipid hydroperoxides via peroxyl radical intermediates. This oxidative reaction is often linked to superoxide anion (O_2_^•−^) generation. Lipid hydroperoxides are subsequently converted into reactive aldehydes, such as malondialdehyde (MDA) and 4-hydroxynonenal (4-HNE), which serve as biomarkers of oxidative stress and cellular damage. The diagram illustrates the Fenton reaction, where ferrous iron (Fe^2+^) reacts with hydrogen peroxide (H_2_O_2_) to generate lipid hydroperoxide, thereby exacerbating lipid peroxidation and promoting ferroptosis. The protective enzyme glutathione peroxidase 4 (GPX4) is depicted as reducing lipid hydroperoxides, thereby inhibiting ferroptosis and preventing cells from oxidative damage.

Lipoperoxidation is one of the main molecular mechanisms involved in oxidative damage to cellular membranes by reactive oxygen species (ROS), targeting lipids with carbon–carbon double bonds, particularly PUFAs [24]. Due to their multiple bonds, PUFAs are especially susceptible to oxidative attack by ROS, such as superoxide anion (O_2_^•−^) and hydrogen peroxide (H_2_O_2_), which are often produced in excess during mitochondrial dysfunction and inflammatory responses. During lipoperoxidation, radicals react with oxygen, generating lipid peroxyl radicals that propagate the chain reaction, ultimately leading to the formation of lipid hydroperoxides. The Fenton reaction, catalyzed by iron, further exacerbates lipid peroxidation by the generation of hydroxyl radicals (OH•) through the interaction of ferrous iron (Fe^2+^) hydrogen peroxide, driving the initiation and propagation of lipid peroxidation, as shown in Figure 1 [25].

The accumulation of lipid hydroperoxides plays a central role in ferroptosis, as these compounds can decompose into toxic aldehydes such as MDA and 4-hydroxynonenal (4-HNE). These aldehydes are highly reactive and can crosslink proteins, nucleic acids, and other cellular components, resulting in cell death [26,27]. LDs, organelles responsible for storing neutral lipids and sterols esters, show a significant increase in number and in MASLD. LDs, along with other ROS-producing organelles, are closely physically associated. For example, NADPH oxidases in mitochondrial membranes and complexes I and III in lysosomes are potential ROS sources in proximity to LDs [28]. LDs act as antioxidant organelles by storing PUFAs in their neutral lipid core, preventing their oxidation [29,30]. Mitochondria associated with LDs, known as peridroplet mitochondria, are abundant in a steatotic liver, likely as a compensatory mechanism to fatty acid overload. This interaction has been linked to the progression of MASLD [31]. PUFAs are particularly susceptible to peroxidation, and lipoperoxidation inside the LDs has been observed in steatotic hepatocytes [32]. Since LDs primarily consist of triacylglycerols and sterol esters, they lack a hydrophilic environment for antioxidant enzymes. Consequently, the only defense against lipid peroxidation within LDs involves lipid-soluble antioxidant molecules such as tocopherol and retinyl esters [33,34]. The assessment of lipid peroxidation in LDs can be achieved using fluorescent probes such as BODIPY-C11, which shifts fluorescence from ~590 nm to ~510 nm upon oxidation. This tool provides valuable insights into lipid peroxidation dynamics in live cells [35].

## 4. Protective Antioxidant Responses in Hepatocytes Against Lipid Peroxidation

The cellular defense against lipid peroxidation involves a variety of antioxidant systems, with Kelch-like ECH-associated protein 1 (Keap1) playing a pivotal role in regulating the antioxidant response. Keap1 controls the activity of nuclear factor erythroid 2–related factor 2 (Nrf2), a key transcription factor. Under conditions of oxidative stress, Keap1 undergoes conformational changes that allow Nrf2 to translocate into the nucleus, where it activates the expression of antioxidant genes [36]. Among these genes are those encoding enzymes such as glutamate-cysteine ligase (GCL), the rate-limiting enzyme in the synthesis of GSH [37]. Glutathione is a critical antioxidant that reduces lipid hydroperoxides through the action of GPX4. GPX4 is essential for detoxifying lipid hydroperoxides, preventing their accumulation and subsequent initiation of ferroptosis. In the absence of sufficient GPX4 activity or adequate GSH levels, lipid hydroperoxides accumulate, leading to the triggering of ferroptosis [38]. This pathway underscores the importance of maintaining redox homeostasis in preventing lipid peroxidation and the associated risk of cell death. Interestingly, an increased proportion of GPX4 positive nuclei was found present in MASLD patients compared to controls [39].

## 5. Ferroptosis and Its Implications in MASLD

Oxidative stress plays a central role in the pathophysiology of MASLD, driving lipid peroxidation of cellular membranes, including LDs, and leading to the formation of reactive aldehydes such as MDA, which can cause cell damage [40]. In high-fat diet (HFD) fed mice, GSH levels are significantly reduced, along with the activity of antioxidant enzymes such as superoxide dismutase (SOD) and catalase (CAT). This imbalance results in increased lipid peroxidation [41]. As mentioned above, GPX4 is a regulator of ferroptosis because of its ability to neutralize lipoperoxidation. Strategies that activate the Nrf2/HO-1/GPX4 pathway can reverse the ferroptosis process in MASLD [42,43]. When GSH is depleted, GPX4 activity also decreases, leading to a rise in lipid peroxides. Furthermore, a dysfunctional cystine/glutamate antiporter system xc− (System Xc^−^) disrupts antioxidant defenses by limiting cystine uptake, exacerbating oxidative stress [44].

A hallmark of MASLD is lipid accumulation within hepatocytes, increasing the availability of fatty acids susceptible to lipid peroxidation [45]. One of the organelles most affected by this lipid overload is the endoplasmic reticulum (ER). Lipid accumulation induces ER stress, which is triggered by the activation of one of the three transducers, inositol-requiring enzyme 1a (IRE1a), PKR-like ER kinase (PERK), and activating transcription factor 6a (ATF6a), culminating in the activation of the unfolded protein response (UPR) [46]. Although LDs offer a protective role, they are insufficient to counterbalance the additional ER stress, leading to UPR activation [47]. Excess intracellular lipids form de novo lipogenesis and contribute to an increase in endogenous saturated fatty acids (SFAs), such as palmitate, which can further promote UPR activation through alterations in phosphatidylcholine species [48]. Elevated levels of SFAs and sterols can stretch the ER membrane, increasing stiffness and causing oligomerization of IRE1 and PERK, activating the UPR [49,50]. This stress disrupts normal LD biogenesis.

## 6. The Role of Myokines on Skeletal Muscle Physiology

Skeletal muscle, a major site of myokine production, plays a crucial role in metabolic regulation and overall health. It contains various cell types, including satellite cells, which are pivotal for muscle growth, repair, and regeneration. Upon muscle contraction, satellite cells are activated, promoting muscle adaptation and hypertrophy, processes that are partly mediated by myokines [51,52]. Key myokines, such as irisin and interleukin-6 (IL-6), enhance glucose uptake and improve insulin sensitivity in skeletal muscle, contributing to metabolic health. These interactions help maintain blood glucose homeostasis and may offer protection against metabolic disorders such as IR and type 2 diabetes. Here, we will discuss the aberrant expression of myokines, their unique characteristics, and the critical role they play in the pathophysiology of MASLD, shedding light on their potential implications for disease progression.

### 6.1. Dysregulation of Myokines Levels Is Associated with MASLD

As discussed, the key hallmark of MASLD is lipid accumulation inside the hepatocytes, which leads to fibrosis and inflammation. Dysregulation of myokine levels significantly contributes to the etiology and progression of MASLD. Myokines regulate metabolic processes, including lipid and glucose metabolism. Conditions of physical inactivity or disabilities can worsen metabolic dysfunction in liver disease [53,54]. However, certain myokines produced during chronic exercise promote β oxidation and glucose uptake, counteracting intrahepatic lipid accumulation. Here, we will present an overview of the most extensively studied myokines, and their association with liver health will be shown.

### 6.2. Myostatin (MSTN)

Myostatin is a dimeric protein from the transforming growth factor (TGF-β) superfamily, composed of two identical 12.4 kDa subunits connected by a disulfide bond. It signals through the activin type II receptors, ActRIIB and ActRIIA, which are expressed in skeletal muscle, heart, and adipose tissue. These receptors trigger intracellular signaling, activating the Smad3/4 transcription factor complex to modulate gene expression [55]. Myostatin is a crucial regulator of muscle mass and metabolic processes, inhibiting muscle growth and promoting adipogenesis. In mice, overexpression of MSTN leads to increased epididymal fat accumulation and reduced cardiac and muscular mass [56]. Conversely, MSTN depletion mitigates age-related increases in adipose tissue, obesity, and diabetes and reduces fat accumulation when consuming a high-calorie diet. Mechanistically, MSTN depletion enhances fatty acid oxidation and lipolysis and promotes brown fat production in white adipose tissue [57].

In the liver, myostatin stimulates HSCs, inducing gene expression program related to fibrosis via activation of the c-Jun N-terminal kinase (JNK) pathway [58]. Additionally, myostatin has an antioxidant effect, as its inhibition enhances AMP-activated protein kinase (AMPK) signaling, which activates glucose-6-phosphate dehydrogenase (G6PD) and the pentose phosphate pathway. This leads to increased NADPH production, higher glutathione reductase activity, and improved antioxidant capacity [59]. MSTN also negatively regulates liver growth hormone by reducing insulin growth factor 1 (IGF-1) levels [60].

It is important to mention that there is conflicting information regarding myostatin levels in liver diseases. On the one hand, myostatin levels are decreased in patients with cirrhosis, and this reduction has been proposed as a predictive biomarker of acute-on-chronic liver failure [61]. On the other hand, elevated serum myostatin levels have been associated with poor prognosis in patients with liver cirrhosis, where increased collagen synthesis is driven by high myostatin levels [62].

### 6.3. Interleukin 6

Inteleukin-6 (IL-6) is a cytokine composed of four α-helices, playing a crucial role in immune signaling. It binds to the IL-6 receptor alpha (IL-6Rα), forming a complex with the signal-transducing receptor gp130 [63]. When gp130 is dimerized by the IL-6 and IL-6Rα complex, it activates the constitutively bound tyrosine kinase JAK1. In hepatocytes, this pathway induces the expression of factors responsible for hepatic acute-phase protein induction, such as hepcidin [64]. Interestingly, while IL6 is a classic biomarker of acute inflammation, exercise also increases plasma IL-6 levels. During sepsis, there is a marked and acute rise of circulating tumor necrosis factor-alpha (TNFα), followed by an increase in IL-6 levels. Studies consistently show that plasma IL-6 is not preceded by a rise in TNFα. It has been consistently shown that plasma IL-6 levels rise during muscular exercise, accompanied by increases in an IL-1 receptor antagonist (IL-1ra) and the anti-inflammatory cytokine IL-10. Additionally, concentrations of chemokines like interleukin 8 (IL-8) and macrophage inflammatory protein-1 alpha (MIP-1α) and beta (MIP-1β) increase, while TNF-α remains unchanged [65]. Notably, chlorogenic acid, a dietary polyphenol, has been reported to reduce hepcidin production by inhibiting the IL-6/JAK2/STAT3 pathway in the liver [66]. This suggests that exercise-derived IL-6 may protect the liver against ferroptosis by inhibiting this pathway. However, MASDL is characterized by high levels of TNFα, which exacerbate the ferroptosis process. In this context, maresin 1 has been shown to inhibit ferroptosis-induced liver injury by suppressing the release of TNF-α and IL-6 [42]. Consistent with this, exercise is a potent stimulus for increasing maresin synthesis [67].

### 6.4. Irisin

Irisin is a peptide consisting of 112 amino acid residues, primarily produced by the cleavage of the fibronectin type III domain containing 5 (FNDC5). It exerts its effects through the integrin αVβ5 receptor, with eHsp90α acting as its cofactor [4]. Due to its antioxidant properties, irisin is considered a hepatoprotective peptide. It regulates crucial cellular processes such as ferroptosis, inflammasome activation, autophagy, mitochondrial fission and fusion, ER stress, and cell death [68]. Irisin promotes the browning of white adipose tissue (WAT) and enhancer thermogenesis by binding to its receptor and activating downstream signaling pathways, including the AMP-activated protein kinase (AMPK) and p38 MAPK [69].

Irisin shows potential as an anti-pyroptosis/apoptosis agent in septic liver injury, as it inhibits apoptosis, NACHT, LRR, and PYD domain-containing protein 3 (NLRP3) inflammasome activation, and nuclear factor-κB (NF-κB) signaling, thereby reducing lipopolysaccharide (LPS) induced liver damage. In LPS-challenged mice, irisin treatment was found to decrease inflammatory responses, ROS generation, and cell death [70]. Additionally, sepsis patients have been shown to exhibit lower serum irisin levels [71]. In septic mice, irisin treatment improved mitochondrial function, increased ATP production, decreased inflammation and ROS levels, and protected against ferroptosis. The protective effects of irisin were associated with GPX4, and these effects were mitigated when irisin’s receptor was blocked [72]. According to this study, exercise reduces hepatic steatosis and fibrosis in NAFLD by increasing irisin levels. Irisin helps limit inflammation by competitively binding to myeloid differentiation protein 2 (MD2) and inhibiting the MD2/Toll-like receptor 4 (TLR4) pathway. The protective effects of irisin on inflammation, fibrosis, and lipid metabolism suggest that exercise may be an effective treatment for MASLD [73].

### 6.5. Fibroblast Growth Factor 21 (FGF21)

FGF21 is a member of the FGFs with endocrine, paracrine, and autocrine functions. It is primarily expressed in the liver, adipose tissue, and skeletal muscle, where its main role is regulating glucose homeostasis and lipid degradation during fasting conditions [74]. FGF21 binds to FGFR1c or FGFR3c receptors, along with the transmembrane co-receptor Klotho, forming a highly regulated protein complex [75]. In hepatocytes, the FGF21/Klotho complex stimulates fatty acid beta-oxidation, gluconeogenesis, and ketogenesis [76]. In adipose tissue, FGF21 promotes lipolysis and glucose [76]. In skeletal muscle, FGF21 plays a crucial role in glucose uptake, muscle remodeling, activating atrophy programs, and mitophagy [77,78]. Acute exercise induces *FGF21* expression in the liver but not in skeletal muscle or adipose tissue [79]. ER stress is one of the triggers of *FGF21* overexpression, mediated through the IRE1α/XBP1 and activating transcription factor 4 (ATF4) pathways or cyclic AMP-responsive element-binding protein H (CREBH) cleavage pathway [80]. These signals activate Nrf2, a critical antioxidant transcription factor, suggesting that FGF21 may serve as a protective myokine, enhancing the antioxidant response in MASLD. Additionally, exercise increases FGF21 sensitivity in adipose tissue by upregulating the gene expression of *FGFR1* and *KLB*, improving metabolic biomarkers such as plasma insulin and triglycerides levels in HFD-fed mice [81]. Overexpression of FGF21 in hepatocytes upregulates *Nrf2* and *Gpx4*, *Slac7a11*, and *Flt* gene expression, improving the cells’ antioxidant capacity. This leads to increased GSH synthesis and reduced lipid peroxidation, highlighting FGF21’s protective role against ferroptosis [82].

### 6.6. Insulin-like Growth Factor

IGF-1 is a peptide hormone with 70 amino acids, primarily produced by the liver. Its expression is stimulated by growth hormone. IGF-1 binds to IGF-1 receptors in skeletal muscle, bone, kidney, and cardiac tissues, with its most well-known functions involving protein synthesis, differentiation, and cell survival [83,84]. The IGF-1/mTOR/p70S6K-1 axis drives an anabolic program within cells [85]. Exercise has been shown to increase IGF-1 gene expression following resistance training in rats [86]. While similar results are observed in humans, a meta-analysis revealed that IGF-1 levels only increase after resistance exercise in individuals over 60 years old but not in younger adults [87]. Additionally, IGF-1 has been reported to play a protective role against liver damage associated with iron overload in a cirrhotic rat model, where IGF-1 treatment prevented increases in MDA, iron stores, and ROS [88].

### 6.7. Brain-Derived Neurotrophic Factor (BDNF)

BDNF is a peptide growth factor classified as a neurotrophin, consisting of 247 amino acids derived from the cleavage of proBDNF. Its receptor, tyrosine receptor kinase B (TrkB), is expressed in both neuronal and non-neural tissues, including skeletal muscle, adipose tissue, and cardiac tissue. The BDNF/TrkB complex activates signaling pathways that regulate synaptic plasticity, energy metabolism, cellular survival, and proliferation [89,90]. Interestingly, BDNF is detectable in human plasma and has been proposed as a biomarker of some neurological diseases [91,92]. Low plasma BDNF levels have been found in individuals with obesity and type 2 diabetes [90], but elevated levels are observed in patients with NAFLD, with the increase being proportional to the severity of the condition [93]. Exercise is one of the stimuli known to increase BDNF plasma levels. For instance, long-term running in mice has been shown to elevate mature BDNF and stimulate neurogenesis in the hippocampus [94]. Another factor that modulates *BDNF* gene expression is β-hydroxybutyrate, a ketone body synthesized by the liver during fasting or exercise. β-hydroxybutyrate is transported via the bloodstream to various tissues, where it stimulates BDNF gene expression in the brain [95]. BDNF also has beneficial effects on the liver, promoting activation of β oxidation of free fatty acids and inhibiting gluconeogenesis through the activation of AMPK in mouse hepatocytes [96]. Some studies suggest that BDNF exerts antioxidant effects, particularly through the activation of Nrf2 in astrocytes. Although inadequate Nrf2 activation in astrocytes may lead to ferroptosis-like cell death in neurons, there is no evidence to establish a direct or indirect antioxidant function in neurons [97].

## 7. Protective Roles of Myokines on Oxidative Stress in the Liver: Molecular Insights

Exercise offers significant benefits in mitigating the effects of MASLD by enhancing both the release and gene expression of myokines. One of the primary benefits of exercise is its ability to increase calorie expenditure while simultaneously activating important metabolic processes like beta-oxidation and gluconeogenesis. Certain aerobic exercise protocols have been shown to alter the architecture of lipid droplets, reducing their size and increasing their contact surface with mitochondria [98]. Regular aerobic exercise is also recommended to improve vascular and metabolic co-morbidities associated with MASLD [99]. However, there is a gap in the evidence regarding the direct impact of specific exercise protocols on reducing liver fat infiltration. For example, a protocol involving 60 min of aerobic exercise per day, 5 days per week, for 15 weeks significantly improved insulin sensitivity, lipid profiles, and the histological score of hepatic steatosis and inflammation in HFD mice. This protocol also enhanced the degradation of hepatic lipid droplets in oxidation markers and increased the protein levels of Nrf2 and GPX4 in HFD-fed mice, indicating enhanced liver antioxidant defenses [100]. In regard to muscle myokines, research showed that a 5-week endurance training in *BDNF* heterozygous knout rat did not alter BDNF/TrkB levels but did improve IL-6 levels, highlighting the potential liver regeneration benefits despite BDNF deficiency [101]. Furthermore, research indicates that hepatic BDNF depletion may actually protect against HFD-induced metabolic disorders by upregulating the PPARα and FGF21 pathways [102]. Recent studies have also underscored the therapeutic potential of the myokine FNDC5/Irisin. It was demonstrated that NAD+-boosting therapy can upregulate FNDC5/Irisin, resulting in positive outcomes like reduced body weight gain, improved hepatic steatosis, insulin resistance, and decreased mitochondrial dysfunction and fibrosis [103]. Chronic irisin treatment in aged mice induces muscle hypertrophy-related gene expression (e.g., MAFbx, MuRF-1, and PGC1α mRNA), alleviating age-associated sarcopenia, enhancing systemic metabolic function, and protecting the liver [104]. Exercise also stimulates the Nrf2/Keap-a/HO-1 signaling pathway in the liver, which has been associated with antioxidant activity, particularly following treadmill training in rats with ethanol-induced liver damage [105]. Additionally, short-term exercise training (30 min for two weeks) has been found to elevate the hepatic expression of antioxidant enzymes, such as catalase (CAT), superoxide dismutase (SOD), and nitric oxide synthase (eNOS), in hyperinsulinemic rats.

The molecular evidence highlights that various exercise protocols offer significant metabolic and liver benefits, although further research is needed to pinpoint the precise mechanisms and optimal regimens for liver fat reduction.

## 8. Conclusions

**Figure 2:**
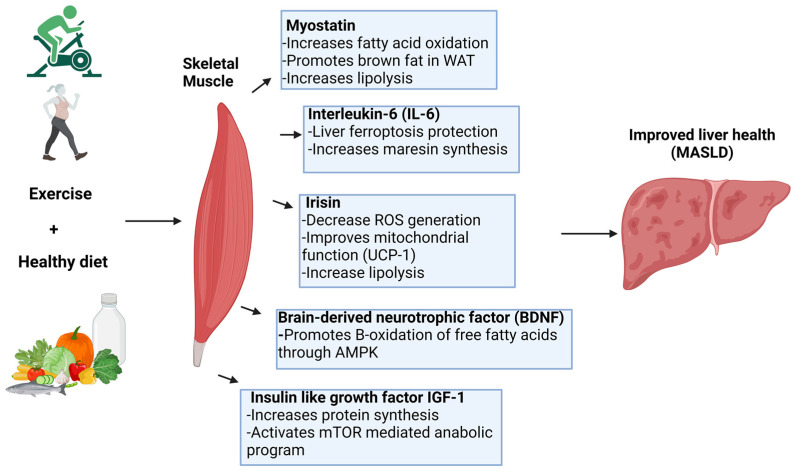
Mechanisms by which myokines improve liver health in MASLD. Myokines released by exercise, including myostatin, IL-6, irisin, BDNF, and IGF-1, promote liver health in MASLD. These myokines enhance fatty acid oxidation, improve mitochondrial function, reduce oxidative stress, and protect against ferroptosis in the liver. Together, these molecular pathways contribute to improved metabolic function and overall liver health in MASLD.

Obesity continues to be a significant global public health issue, with metabolic dysfunction-associated steatotic liver disease (MASLD) as one of its primary consequences. MASLD, defined by hepatic fat buildup without evident symptoms, currently has limited therapeutic approaches. Nonetheless, lifestyle alterations, especially physical activity, have demonstrated encouraging outcomes in slowing and even reversing the progression of liver damage due to MASLD. Specifically, we provided evidence of how physical activity inhibits lipid droplet formation and subsequent oxidation in the liver, as well as the impact of muscle-derived myokines on the liver (Figure 2).

We provided evidence from preclinical and clinical research that myokines improve cellular processes such as ER stress, mitochondrial dysfunction, and glucose mismanagement in the liver. This slows down the activation of fibrotic programs associated with the progression of liver damage and can facilitate the reversal of MASLD. However, more research is needed as the literature is confined to a specific subset of myokines. Despite the limited number of myokines that have been investigated, they have provided significant insights into the intricate biological mechanisms that slow and reverse MASLD progression.
